# Effect of artifacts upon the pressure reactivity index

**DOI:** 10.1038/s41598-022-19101-y

**Published:** 2022-09-06

**Authors:** Martin Rozanek, Josef Skola, Lenka Horakova, Valeriia Trukhan

**Affiliations:** 1 Department of Biomedical Technology, Faculty of Biomedical Engineering, Czech Technical University in Prague, Nam. Sitna 3105, 272 01 Kladno, Czech Republic; 2 Department of Anaesthesiology, Perioperative and Intensive Care Medicine, Masaryk Hospital in Usti nad Labem, Faculty of Health Studies, J. E. Purkinje University in Usti nad Labem, Usti nad Labem, Czech Republic; 3 Faculty of Medicine in Hradec Kralove, Charles University in Prague, Prague, Czech Republic

**Keywords:** Neuroscience, Neurology

## Abstract

The pressure reactivity index (PRx) is a parameter for the assessment of cerebrovascular autoregulation, but its calculation is affected by artifacts in the source biosignals—intracranial pressure (ICP) and arterial blood pressure. We sought to describe the most common short-duration artifacts and their effect on the PRx. A retrospective analysis of 935 h of multimodal monitoring data was conducted, and five types of artifacts, characterized by their shape, duration, and amplitude, were identified: rectangular, fast impulse, isoline drift, saw tooth, and constant ICP value. Subsequently, all types of artifacts were mathematically modeled and inserted into undisturbed segments of biosignals. Fast impulse, the most common artifact, did not alter the PRx index significantly when inserted into one or both signals. Artifacts present in one signal exceeded the threshold PRx in less than 5% of samples, except for isoline drift. Compared to that, the shortest rectangular artifact inserted into both signals changed PRx to a value above the set threshold in 55.4% of cases. Our analysis shows that the effect of individual artifacts on the PRx index is variable, depending on their occurrence in one or both signals, duration, and shape. This different effect suggests that potentially not all artifacts need to be removed.

## Introduction

The pressure reactivity index (PRx) is one of the recognized parameters for the assessment of cerebrovascular autoregulation, with a more than 20-year history in neurointensive care. PRx is determined as the moving-window Pearson’s correlation coefficient between arterial blood pressure (ABP) and intracranial pressure (ICP). There is a strong association between PRx-detected impaired cerebrovascular autoregulation (CAR) and unfavorable outcomes following severe traumatic brain injury (TBI)^1^. Patients with impaired autoregulation may also profit from a different treatment strategy. Moreover, in an experimental setting, PRx possibly allows for calculation of the optimal cerebral perfusion pressure^2,3^. However, it is a very noisy parameter with several limitations^4^.

The reliability of the PRx calculation is crucial for its potential clinical use. In a pilot study by Dias et al*.*^5^, the quality of the captured signals and calculated PRx were always inspected before the recommended optimal cerebral perfusion pressure (CPPopt) was considered by the attending physician.

The presence of artifacts in source biosignals poses a significant challenge. Manual artifact removal is laborious and delayed, which limits PRx use as a real-time clinical tool. The software ICM+ (Cambridge Enterprise Ltd, Cambridge, UK), commonly used for research in neurocritical care, has an embedded tool for the detection of aberrant signals^6,7^. The authors of this software recommend treating artifacts as missing values rather than substituting them with the results of calculations from surrounding values^6^. Embedded online artifact detection is based on the identification of signal values outside the set range or vanishing of the pulse wave amplitude in signals with a pulsatile character. The artifact detection algorithm can also be personalized by the user within the secondary calculations^6,7^.

Several attempts have been made to identify the sources of the artifacts. Apart from the artifacts originating from disconnection of the monitors and from technical issues, there are several situations common in an intensive care unit that can distort the waveforms and values (patient positioning, coughing, etc.). Previous studies have investigated the pattern^8^ and the origin^9^ of the artifacts in the arterial blood pressure signal; some studies have also attempted to detect them^10–13^ or even to eliminate them^9,14^, mainly using artificial intelligence tools and neural networks.

Automated artifact detection and removal from both ICP and ABP signals in patients with traumatic brain injury was proposed by Lee et al*.*^15^. Their system based on a stacked convolutional autoencoder and convolutional neural network was able to detect artifacts with high sensitivity (97.3% for ABP and 96.2% for ICP). The study also proved the effect of artifact removal on the incidence of four major clinical events in TBI patients: systemic hypotension, intracranial hypertension, cerebral hypoperfusion, and impaired cerebrovascular reactivity. Among these clinical events, poor cerebrovascular reactivity (defined as PRx > 0.3) was the most frequently observed before and even after artifact removal. The authors speculated that the real incidence of these events might be lower in biosignals without artifacts, which can have serious treatment consequences.

The aim of this study was to investigate the effect of common artifacts in ABP and ICP signals on the calculation of PRx. Specifically, the identified artifacts were further analyzed to evaluate how relevant it is to remove these segments from the signals and how they alter the PRx value.

## Methods

### Type of study

This study was a retrospective analysis of high-frequency multimodal monitoring data records of patients with intracranial pressure monitoring from an anonymized database. Monitoring of these brain modalities was a part of an ongoing project of the TBI registry. Extracted data were fully anonymized, and no data on patient identifiers were available. All methods were performed in accordance with the relevant guidelines and regulations.

### Data

In total, 935 h of anonymized data from seven patients with acute brain injury and invasive intracranial pressure monitoring were analyzed. The average length of recording was 133 h (range 48–256 h). The data were recorded in the Neurointensive Care Unit at the Department of Anesthesiology, Perioperative and Intensive Medicine, Masaryk Hospital in Usti nad Labem, Czech Republic. The datasets comprised only variables of vital sign parameters, and no patient characteristics (e.g., sex, age, date of admission, diagnosis, outcome) were available.

### Equipment

All patients had continuous synchronized data collected via ICM+ software (version 8.6, Cambridge Enterprise Ltd., Cambridge, UK): ABP, ICP, electrocardiography and carbon dioxide waveforms. The physiological parameters were monitored using a Carescape B850 vital signs monitor (GE Healthcare, Helsinki, Finland). All signals were sampled at 200 Hz. The pressure transducer for APB measurement was placed at the level of the tragus^16^. ICP was monitored with a multimodal sensor (Neurovent PTO, Raumedic AG, Helmbrechts, Germany) measuring ICP, brain tissue partial pressure of oxygen, and brain temperature and logged by a Raumedic MPR 2 logO monitor (Raumedic AG, Helmbrechts, Germany).

### Data analysis

The primary data analysis was conducted using ICM+ software. The PRx index was calculated as linear (Pearson’s) moving correlation coefficients between 30 past consecutive 10 s-averaged values of ICP and ABP using a 60 s moving window. For every 300 s, the 30 averaged values of arterial blood pressure and intracranial pressure were available^4^.

First, all available raw data, sampled with the frequency of 200 Hz, were manually inspected for artifacts. The recognized artifacts were cataloged and described in terms of their shape, duration, incidence, presence in one or both signals, and the percentage of increase or decrease in the signal amplitude (i.e., ABP change from 100 to 125 mmHg was considered a 25% amplitude rise).

Subsequently, data segments including undisturbed blood and intracranial pressure waveforms lasting at least 10 min without any artifacts were identified. The criteria for these segments were ICP 7–15 mmHg with a physiological trifold waveform, mean arterial pressure 90 ± 5 mmHg and PRx below 0.3. In total, twenty 10-min segments fulfilling all these criteria were selected from all available data and used for further analysis.

All selected undisturbed signal segments were exported using csv format into MATLAB (version R2019a, MathWorks, Natick, Massachusetts, USA). In this software, the calculation of the PRx index was conducted in the same standardized manner as in the ICM + software. The most common artifacts were modeled in MATLAB software and inserted into the undisturbed segments of ABP and ICP waveforms. The lengths and amplitudes of individual artifacts were modeled based on the native artifacts.

Into each of these undisturbed segments at the time point of 4 min 1 s, one modeled artifact was inserted. The artifacts were inserted into either one or both pressure signals. Following that, the PRx index was calculated in a standard way. The individual artifacts modeled in MATLAB software are depicted in Supplementary Material 1.

For each of these twenty undisturbed segments, six pairs of PRx indexes were calculated before and after artifact insertion (see Supplementary Fig. 2, S1). In the event of the presence of artifacts lasting less than one minute, just five out of six PRx coefficients were affected by the artifacts. For artifacts lasting more than one minute, all six coefficients were affected, as the artifact had an impact on more segments (see Supplementary Fig. 3, S1). From each segment with an inserted artifact, one PRx value was randomly chosen. This randomly chosen PRx index was then paired with a PRx index from a corresponding part of the undisturbed segment. This process was then repeated for all twenty segments and all defined artifacts.

The first part of the analysis evaluated the main characteristics of the artifacts in the measured signals and consequently their effect on the PRx value. In the second part, the increase in PRx over the limit value was calculated. PRx threshold of 0.3 was considered clinically relevant^1,4,17,18^.

The sets of computed PRx were tested for normality of distribution using the Lilliefors test. Some sets of PRx coefficients lack the normal distribution. Therefore, Wilcoxon signed-rank test was performed on all data sets. A p-value of < 0.05 was considered significant. The results are expressed as median [interquartile range].

### Compliance with ethical standards

The study was approved by the Ethics Committee of the Masaryk Hospital in Usti nad Labem, Czech Republic (Reference number 298/1). The need for patient consent was waived.

## Results

### Observed artifacts

**Figure 1:**
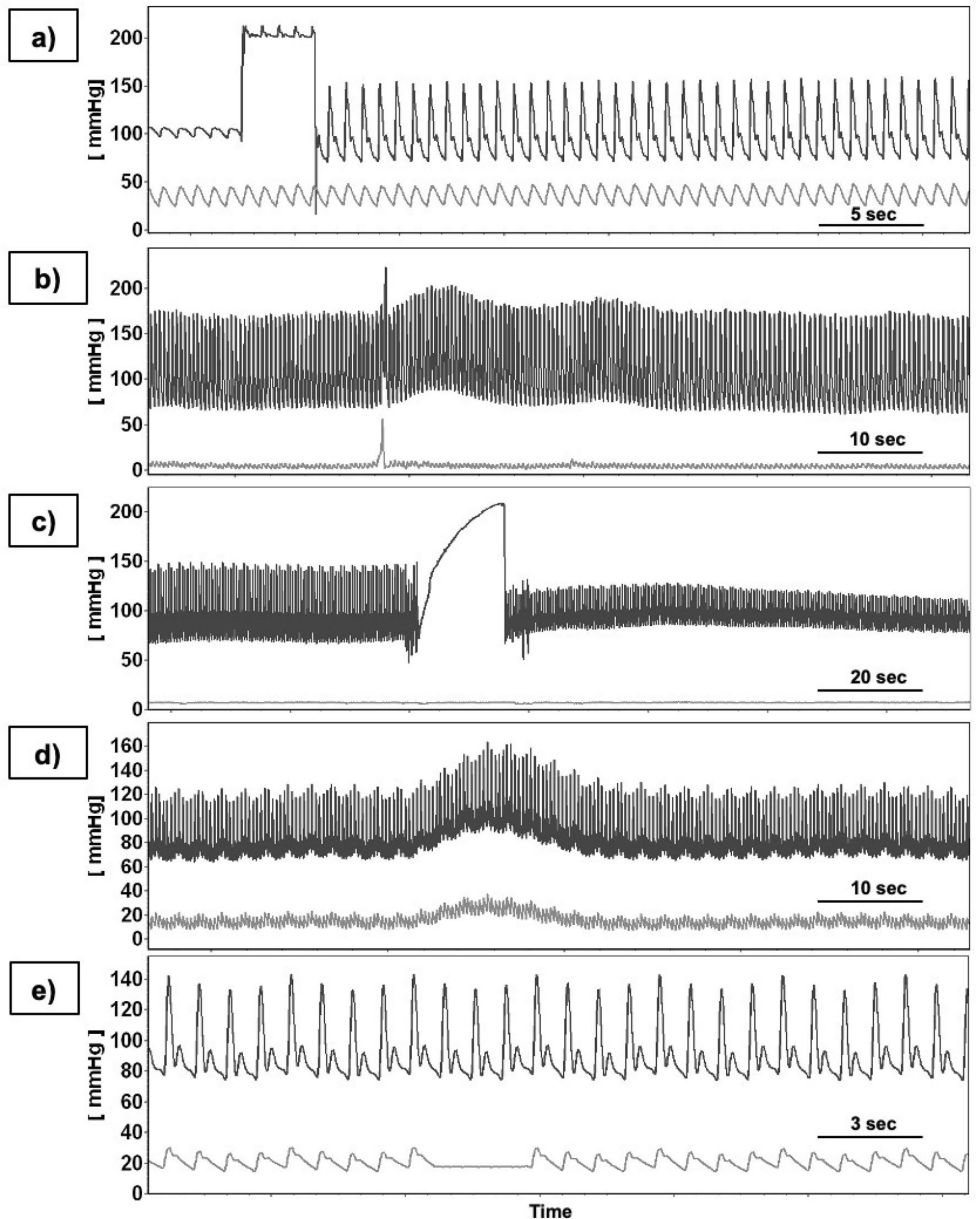
Identified shapes of artifacts: (**a**) rectangular, (**b**) fast impulse, (**c**) saw tooth, (**d**) isoline drift, (**e**) constant value.

The whole dataset contained two main groups of artifacts, described as “stereotyped” or “complex.” Stereotyped artifacts could have been described in terms of their shape, duration and amplitude. Unlike this, complex artifacts lacked the above mentioned properties and could not be described and characterized for the analysis used in this study. The stereotyped artifacts were categorized according to their shape, as rectangular artifacts, fast impulses, saw tooth-shaped signals, isoline drifts, and constant ICP values (see Fig. 1). In Table 1, the characteristics of the artifacts are presented: the occurrence in the signals of ABP and ICP and parameters of the signals modeled based on these observed artifacts. The average number of artifacts per patient in a 24 h signal segment was 166 with a standard deviation of 41. The lengths and amplitudes of individual artifacts were modeled based on the common duration and shape of the native artifacts. For this reason lengths and amplitudes of the different types of artifacts vary.

**Table 1:** Observed artifacts, their occurrence in arterial and intracranial pressure waveforms and parameters of simulated artifacts (duration and amplitude change).

Type of artifact	Affected signals				Parameters of modelled artifacts	
	ABP	ICP	ABP and ICP	Proportion of artifact (%)	Duration (s)	Amplitude rise (%)
Rectangular	X		X	34.7	4; 15; 30; 60	25; 50 ;75; 100
Fast impulse	X		X	30.0	0.04	25; 50; 75; 100; 125
Saw tooth	X		X	9.5	30; 45; 90	30; 60
Isoline drift			X	3.2	15; 30; 60; 120	15; 30
Constant value		X		22.7	4	Constant value of ICP 8 mmHg

The most common artifact appearing solely in the arterial blood pressure signal with a normal ICP waveform was the artifact with a rectangular shape. In some cases, this artifact was present in both signals. The duration of the rectangular artifact was on average 4 s, but a longer time span was also observed.

The fast impulse—lasting only 0.04 s—was frequent in both signals. Only in a minority of data recordings did the fast impulse affect either ABP or ICP signals.

In contrast to the ubiquitous fast impulse and rectangular artifact, the saw tooth signal was recorded only in just a few data segments. It was observed mainly in arterial line signals but also simultaneously in both signals.

An artifact that always occurred in both pressure waveforms was the simultaneous isoline drift. Compared to previously mentioned artifacts, this artifact belonged to the longest noncomplex artifacts, lasting up to 2 min. The identification of this artifact was based on an observation of simultaneous amplitude increases in both ABP and ICP with no apparent periodicity. As this artifact is close to a physiological waveform behavior, the doubtful signal segments were discussed with two clinicians experienced in neurocritical care. Only in cases when both clinicians agreed on the artificial origin of the segments, the signals were evaluated as isoline drifts. In total, 24 doubtful drifts were initially identified and both clinicians agreed on an artificial origin in 4 cases.

The single artifact present only in the intracranial pressure waveform was the constant value of ICP, lasting 3 to 4 s. The displayed constant value was within the pressure range recorded before this artifact appeared but with a complete loss of the typical trifold waveform.

### Artifact effect analysis

**Figure 2:**
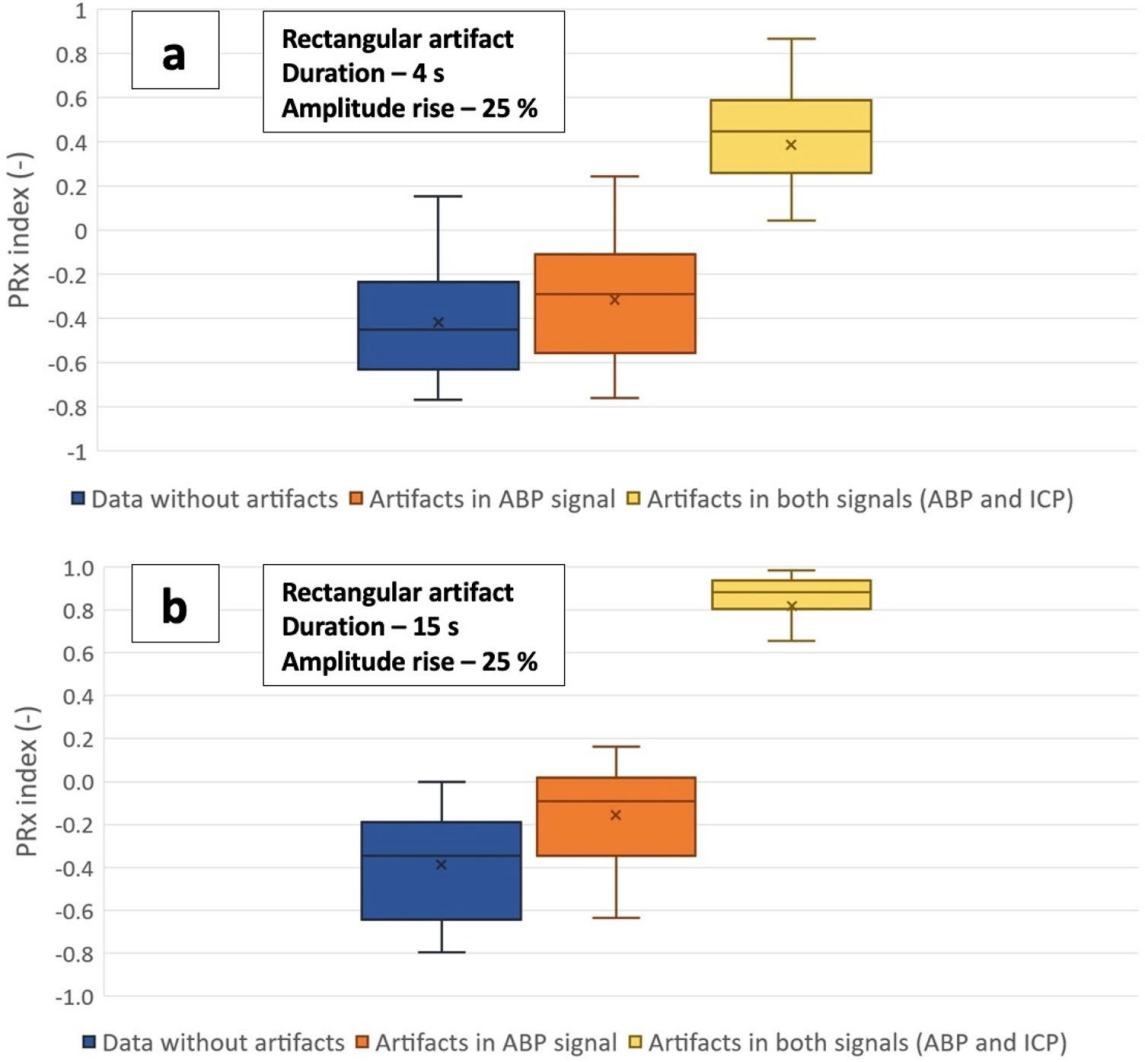
The effect of the rectangular artifact on PRx value. Even the shortest artifact of this shape present in both signals causes a significant change in the PRx index; when the artifact is prolonged to 15 s, the PRx index approaches 1.0. Box-and-whisker plots representing the median value, with 50% of all data falling within the box (interquartile range). The "whiskers'' extend to the minimum and maximum. Cross inside the box represents the mean value. (**a**) Box plots depicting the effect of rectangular artifacts lasting 4 s with an amplitude rise of 25% in the arterial blood pressure waveform and in both waveforms. (**b**) Box plots depicting the effect of rectangular artifacts lasting 15 s with an amplitude rise of 25% in the arterial blood pressure waveform and in both waveforms.

Four artifacts were inserted into both signals—arterial and intracranial pressure: the rectangular artifact, the fast impulse, the saw tooth artifact and the isoline drift. Even the shortest and smallest rectangular artifact lasting 4 s with only a 25% increase in amplitude caused an increase in PRx over 0.3 (PRx 0.45 [0.26–0.59]) in more than half of the samples (55.4%). For longer artifacts lasting 15 s and more, the PRx reached the set threshold in all cases—very often the correlation coefficient was close to 1.0 (for 15 s long artifact: PRx 0.88 [0.81–0.93]) (Fig. 2). This behavior was even more pronounced in the presence of the saw tooth artifact, in which the rise of the amplitude of 30% caused a surge of the PRx index to 1.0 in all cases, regardless of how long the artifact was (30, 45, or 60 s). Compared to the previous two artifacts, the isoline drift caused a significant change in the PRx index only when the drift lasted at least 1 min and the baseline ascended by nearly a third; in these cases, more than one-fifth of the PRx coefficients (21.6%) reached a value above 0.3. The most common artifact—the fast impulse—did not show a statistically significant change in the PRx index for all simulated amplitudes when inserted only into ABP or into both signals.

The artifacts inserted only into one of the signals showed an unalike behavior. The rectangular artifact in ABP revealed a set threshold value of PRx in maximum at 4.3% (30 s duration with amplitude rise 75–100%); even in the longest and tallest artifact (60 s long with 100% amplitude rise), the occurrence of the critical PRx value was in 2.9%. A similar behavior was observed for saw tooth artifacts, with the highest increase to critical PRx value in just 4.3% of the cases. These results are different compared to the presence of artifacts in both signals, where the surge of a PRx index over 0.3 occurs in all cases (see Supplementary Table S1, S2).

The only artifact simulated solely in the intracranial pressure was a constant value of ICP for 4 s, where the change in the PRx index did not reach the critical value and the change in the PRx index was not statistically significant (p = 0.16).

## Discussion

The main finding of this study is that the effect of artifacts on PRx is variable depending on their shape, duration, and presence in one or both signals. The most common artifact—the fast impulse—does not have a significant effect on the calculated PRx value, even when present in both signals—ABP and ICP. Although this artifact is generally easily detectable by manual inspection or by automatic artifact detection systems, the negligible effect on the PRx calculation suggests that removal of this artifact is not critical for signal analysis.

**Figure 3:**
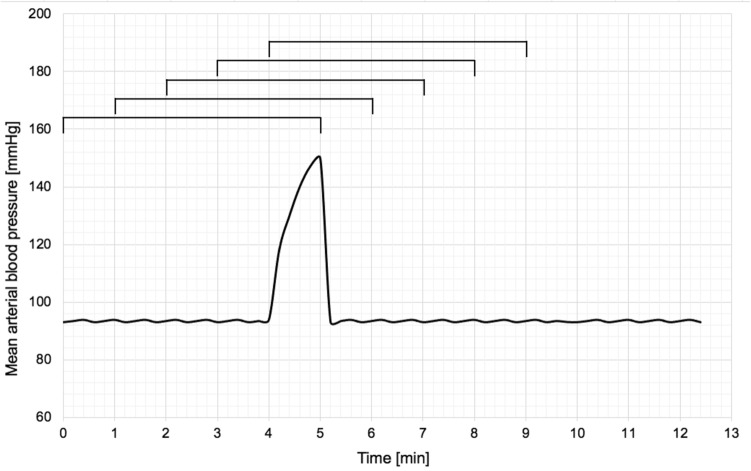
The effect of an artifact on a moving window calculating the PRx index. One single artifact affects five consecutive PRx calculations.

On the other hand, even a 4 s-long rectangular artifact with a 25% rise in amplitude present in both signals significantly affects the PRx value. With increasing duration of the artifact, reaching 15 s or more, the PRx in all studied cases reached nearly 1.0. Moreover, due to the length of the moving calculation window (300 s), even this 4-s-long artifact in both signals affects the PRx calculation for 5 min (see Fig. 3).

The presented results show that despite the 10-s averaging window, an artifact in both signals is prone to change the PRx towards values over 0.3, suggesting disturbed cerebral autoregulation, which can have significant consequences for treatment strategy. For an alteration of the PRx index, not only the amplitude but also the duration of the artifact in the source biosignals is important.

When artifacts occur in both signals, they can alter the PRx even if the artifacts last only a couple of seconds with an amplitude increase of tens of percent. The authors hypothesized that the larger the area under the curve (AUC) of the artifact, the larger the impact on the correlation coefficient calculation. However, in the presence of the artifact in both signals, the importance of the area of the artifact is less pronounced. Importantly, except for the fast impulse, an artifact in both signals routinely changes the PRx towards 1.0 (Supplementary Fig. 4, S1), which is clinically consequential. Thus, these artifacts are worth removing.

Some of the artifacts can be removed using an automatic or a manual tool embedded, for instance, in the ICM+ software. However, even the artifacts discovered by the automatic removal tools are usually reviewed by a researcher or a clinician which is a laborious work and suitable mainly for a post hoc offline analysis only.

Differentiation of the artifact from a physiological signal is challenging. Pasma et al*.*^13^ compared the algorithmic and manual annotation of artifacts in arterial blood pressure signals during anesthesia. There was a significant discrepancy among the direct observation of the artifacts and the retrospective analysis or the detection using machine learning algorithms. Although the data presented in this study were analyzed only retrospectively and without any clinical information about potential sources of artifacts, several artifacts necessitated two experienced physicians to decide about their artificial or physiological origin.

The results support the proposed concept of automatic online artifact detection systems^15^. Such a system allows not only the detection but also the bridging of these segments, so the final reliable results of advanced calculations are presented to the clinician at the bedside.

Lee et al*.*^15^ suggested that all segments with artifacts, regardless of whether they occur in ABP, ICP, or both signals, should be simultaneously removed due to their potential effect on the reliability of the composite parameters, such as cerebral perfusion pressure (CPP) and PRx. However, our results propose that some of the simple artifacts do not affect the PRx values significantly; thus, their removal is not crucial. Moreover, a global removal of signals can cause loss of important clinical information.

The 'clarity' of the signals and the calculated parameters used in the management of patients with intracranial hypertension can significantly increase the prognostic potential and future clinical value of these parameters. Lee et al*.* also hypothesized that some clinical events, such as intracranial hypertension and cerebral hypoperfusion, might be in reality less frequent, as seen in biosignals following artifact removal. Thus, the effect of the artifacts might be greater than previously expected^15^.

For this study we have classified artifacts as “stereotyped” or “complex”. Stereotyped artifacts could have been described in terms of their shape, duration and amplitude and thus modeled in MATLAB software for a subsequent analysis. Unlike this, complex artifacts lacked the above-mentioned properties and could not be described and characterized for analysis method used in this study.

The complex artifacts are likely of multifaceted origin and detailed investigation without well clinically annotated signal records is deemed impossible by the study team. We hypothesize, that they—at least in part—origin from the nursing care (patient suctioning, positioning or overall care) or from physiological phenomena like coughing. Additionally, the goal was not to categorize the artifact types according to their cause but to find some common waveform patterns among them. Studies with a systematic recording and assessment of the potentially disturbing situations which may lead to artifact generation should help us better understand the causes of these artifacts. The authors propose that finding the cause of an artifact is less important than properly identifying it and treating it in data management.

Moreover, this study suggests that certain types of artifacts can have a significant effect on PRx calculation, thus possibly affecting its value as a research or clinical tool. Various strategies to deal with artifacts have been proposed, from retrospective manual removal, simple logic filtering to machine learning or artificial intelligence approach^12–15^. Based on available evidence, no universal strategy can be proposed. Artifact treatment remains a significant challenge for future research in this field.

The current study has several limitations. First, only raw data were analyzed, and detailed information about the patients (e.g., their age, sex, diagnosis, outcome, etc.) was not available. This makes the correlation between the effect of the artifacts and the outcome impossible. However, this study was focused solely on the effects of artifacts on PRx itself, which does not require any further patient data.

Second, the modeled artifacts were applied on undisturbed segments where cerebrovascular pressure autoregulation (CAR) was still preserved to some extent, as documented by the PRx values below 0.3. The effect on signals with ICP over 15 mmHg was not assessed. On the other hand, patients with intracranial pathology, yet with preserved CAR, are also vulnerable to incorrect treatment potentially based on artifact-affected parameters. However, further research of artifact effect on ICP signals in case of elevated intracranial pressure or pathological morphology of ICP waveform is needed.

## Conclusions

Our study shows that the effect of artifacts on PRx calculation is variable. While the most frequent artifacts—fast impulses—are easily detectable and can be automatically removed, they do not significantly alter the value of PRx. Other artifacts that significantly affect PRx are mainly the artifacts present in both signals—ABP and ICP—except for fast impulse. Even four-second-long rectangular artifacts that occur in both signals with a small amplitude rise cause substantial changes in PRx above the threshold value of 0.3.

Most of the artifacts analyzed in this study affect the PRx value. This study underlines the need for careful artifact treatment prior PRx calculation, as certain types artifacts can have significant impact on PRx value, possibly leading to faulty data interpretation. However, the present results suggest that not all artifacts have the same effect on the PRx calculation, and potentially, not all of them have to be removed via laborious work.

## Supplementary Information

Supplementary Figures.

Supplementary Table S1.

## Data Availability

The datasets analyzed during the current study are available from the corresponding author on reasonable request.
