# Supplementary material for: Effect of artifacts upon the pressure reactivity index
Source: Sci Rep. 2022 Sep 6;12:15131. doi: 10.1038/s41598-022-19101-y (PMC9448724; doi:10.1038/s41598-022-19101-y)
Supplement: Supplementary file 1 — Supplementary Figures. [file 41598_2022_19101_MOESM1_ESM.docx]

Effect of Artifacts upon the Pressure Reactivity Index

- Supplementary material 1

Martin Rozanek, Josef Skola, Lenka Horakova, Valeriia Trukhan

__________________________________________________________________________

This supplementary material contains details regarding methodology of data analysis and modeling of artifacts.


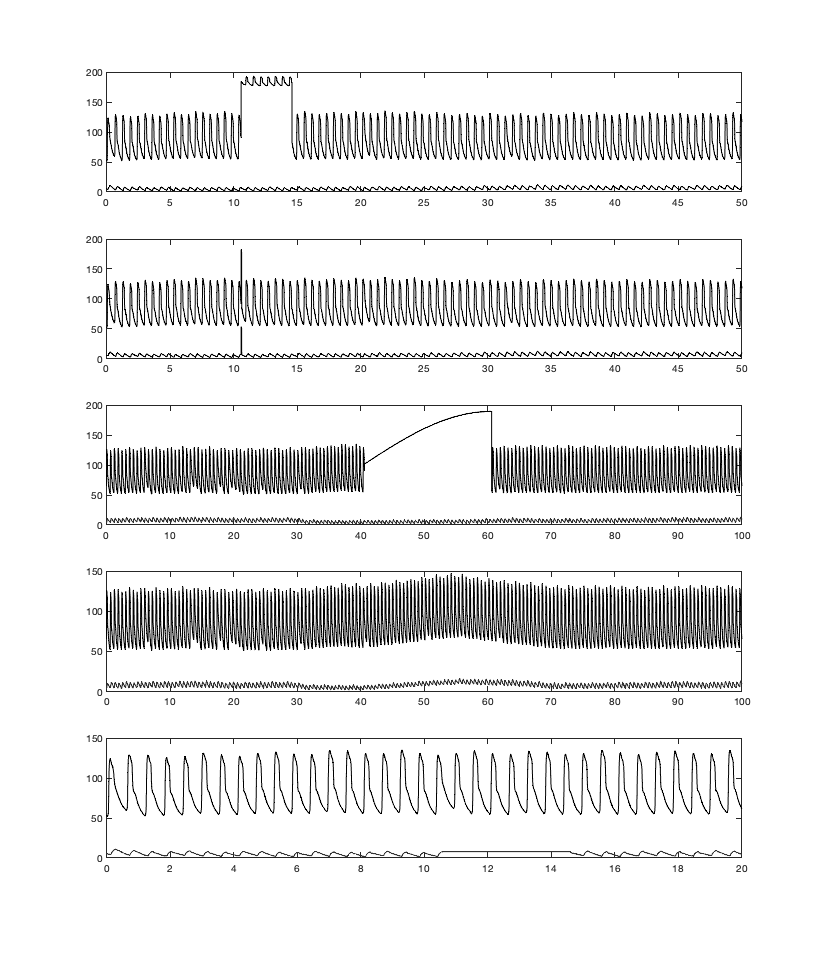


**Supplementary figure 1.** Artifacts modeled in MATLAB software a) rectangular, b) fast impulse, c) saw tooth, d) isoline drift, e) constant value.

**Supplementary figure 2.** Diagram of steps of PRx calculation from undisturbed signal segments and following an insertion of modeled artifacts.

**
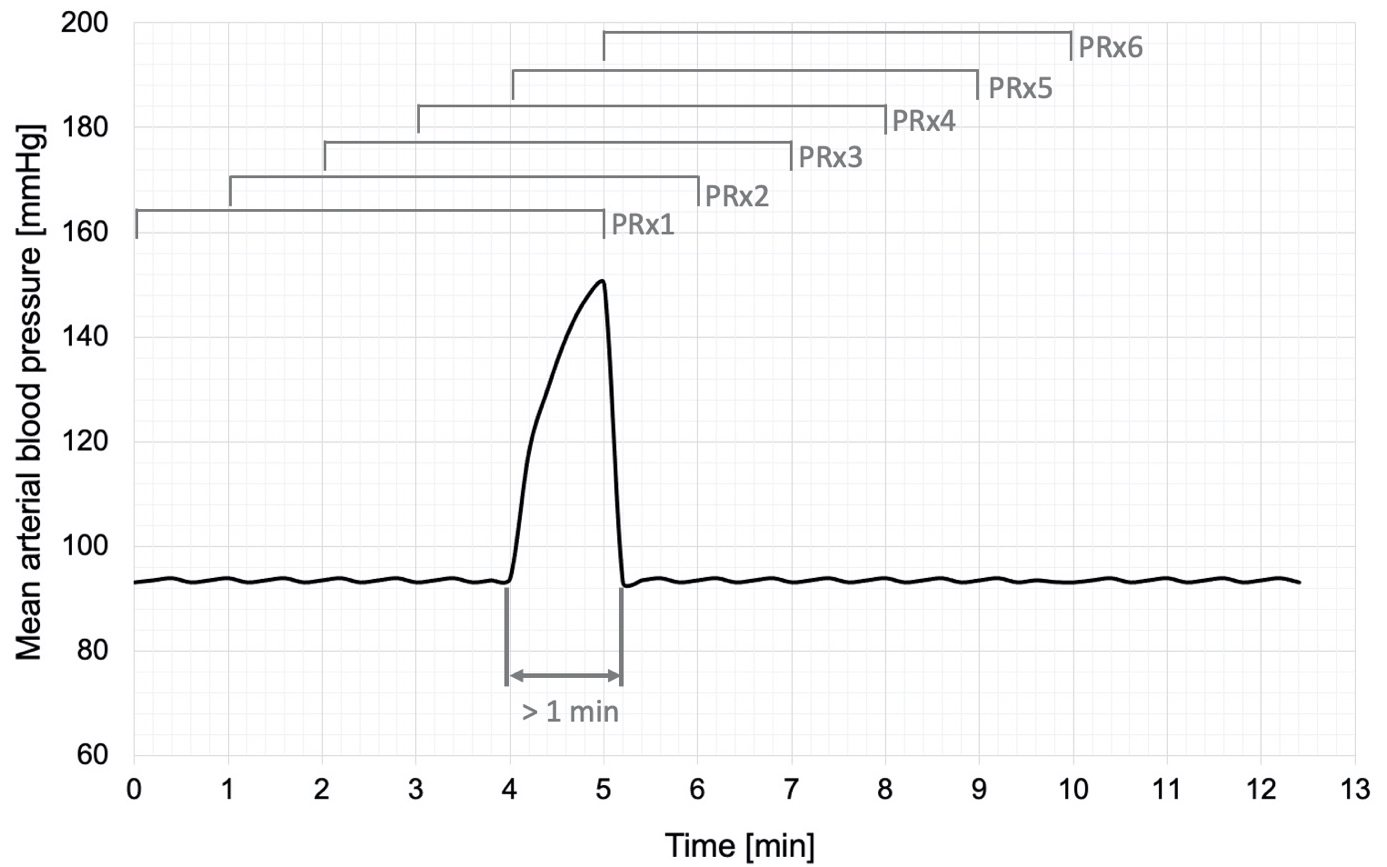
**

**
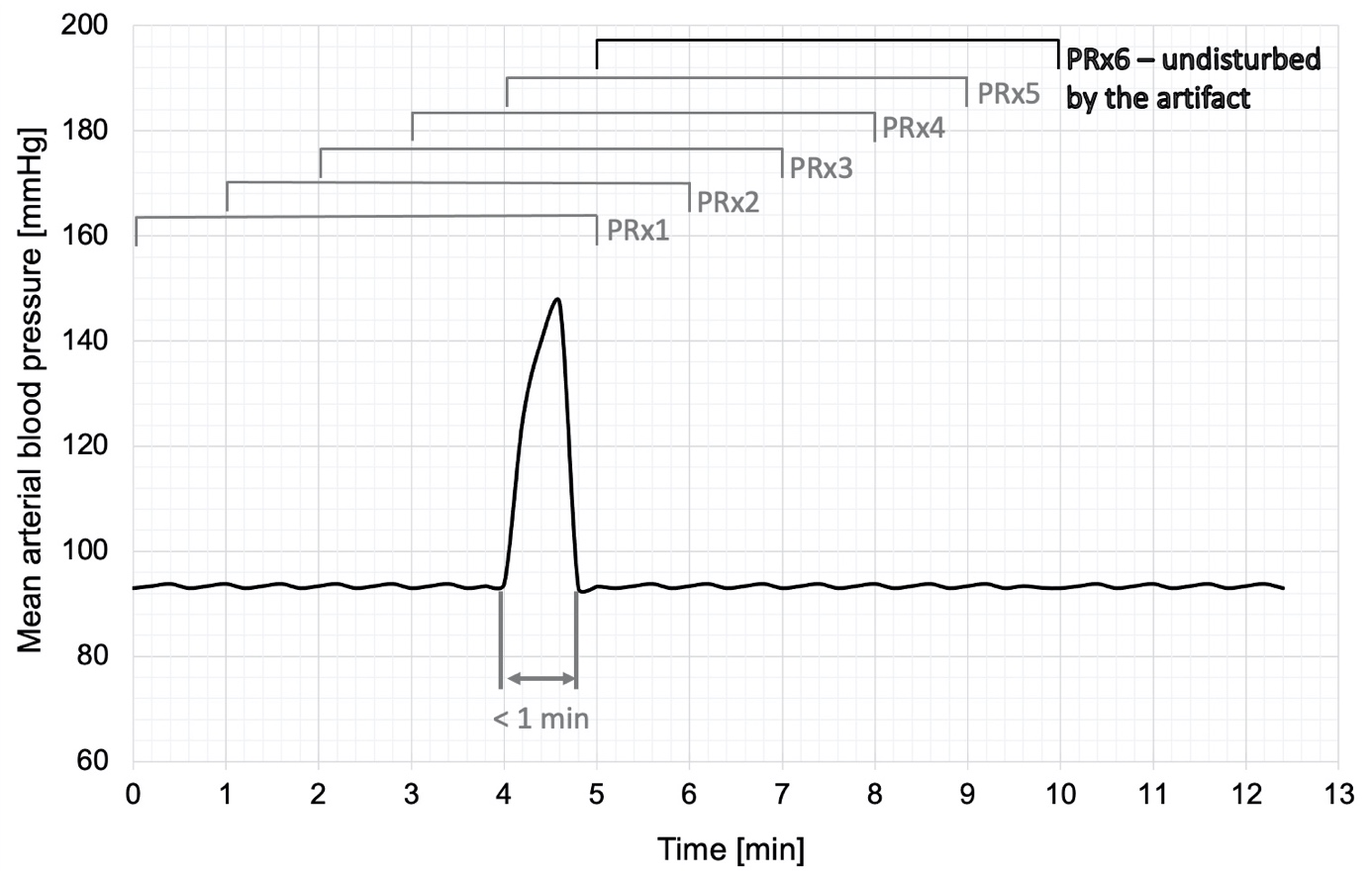
**

**Supplementary figure 3.** Affected PRx coefficients by artifact inserted in ABP signal. Length of artifact > 1 min (upper figure) and length of the artifact < 1 min (bottom figure).

**
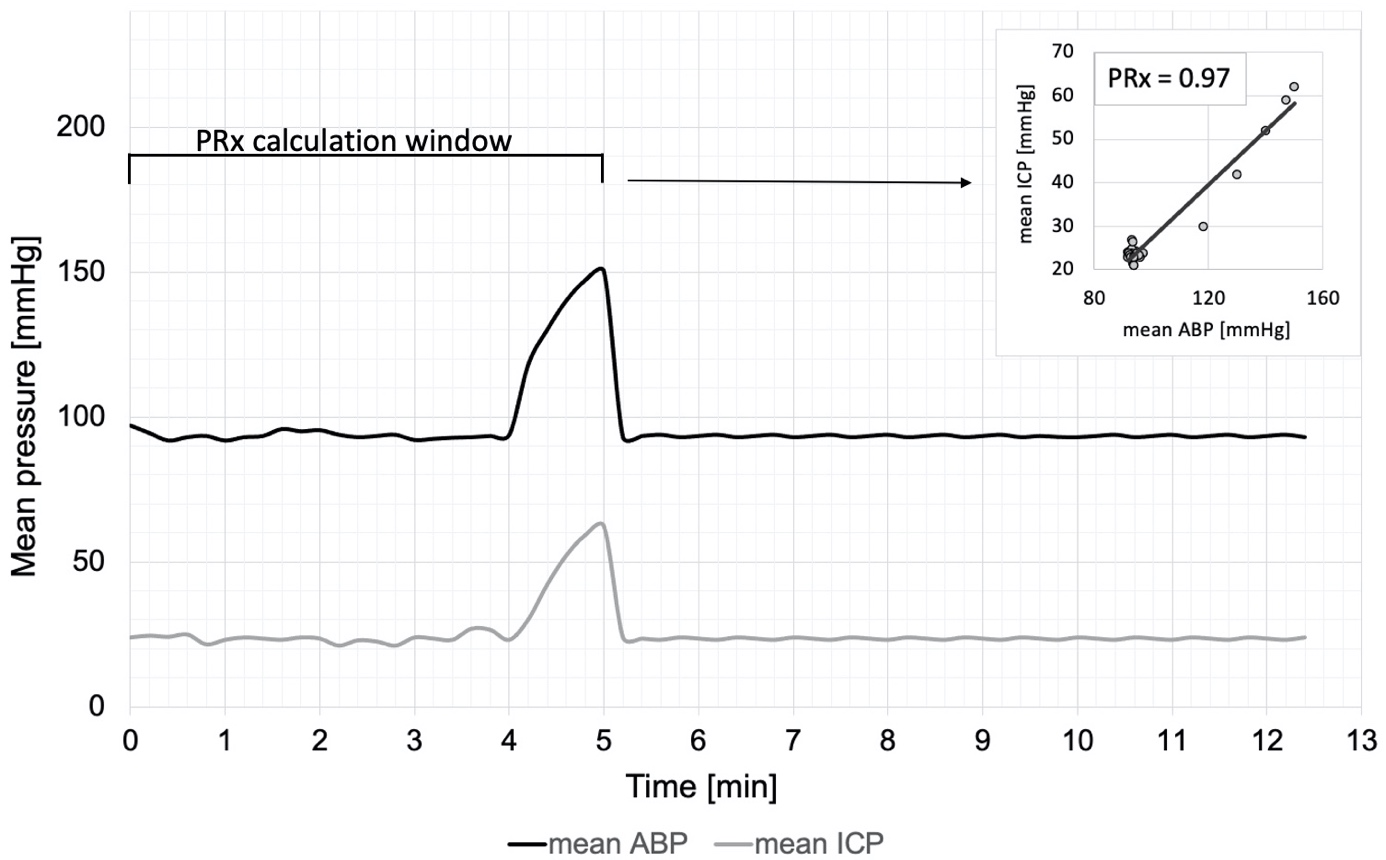
**

**
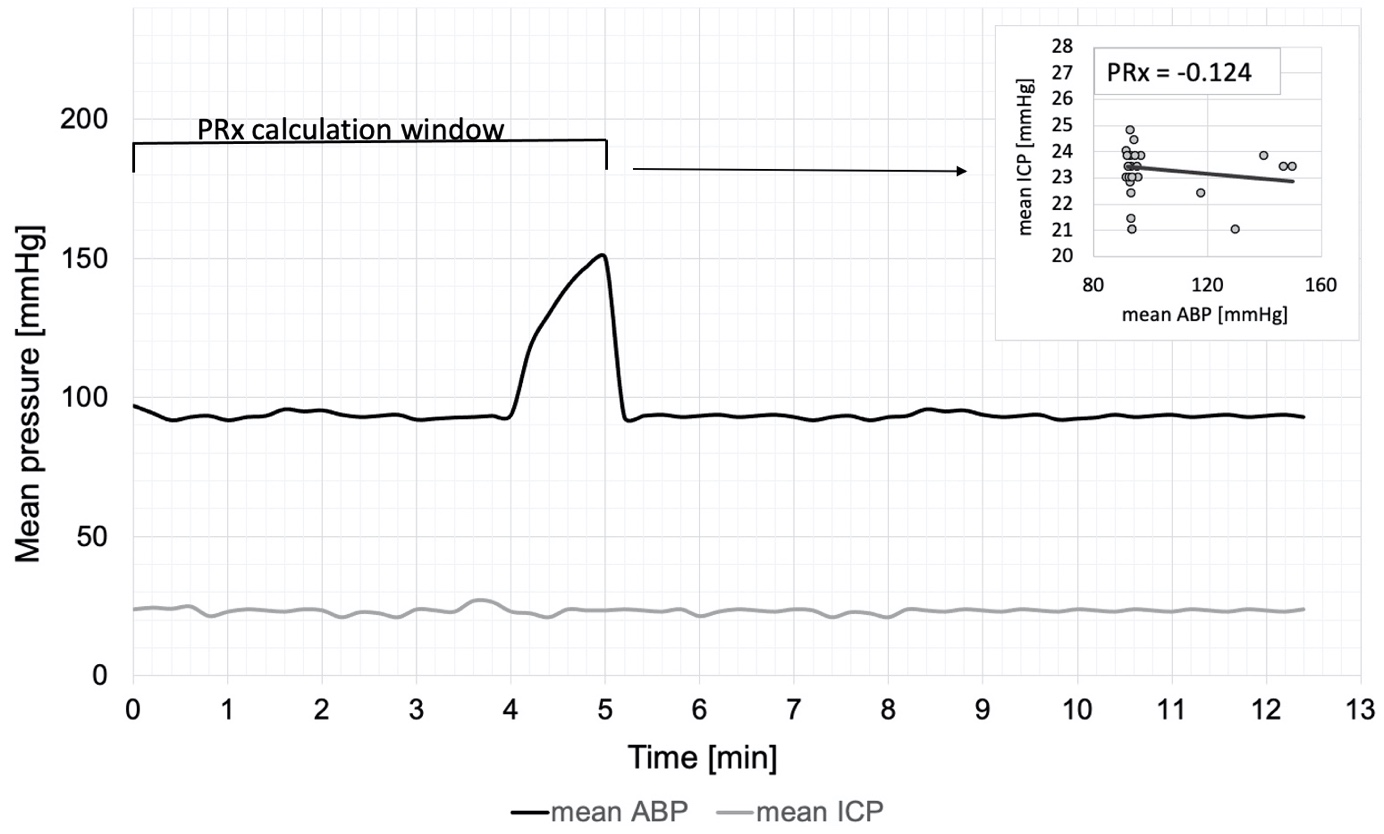
**

**Supplementary figure 4.**

The effect of the artifact placed into ABP and ICP signals (upper figure). The increased values of ABP and ICP at the same time due to the artifact placement cause the increase of PRx close to +1.

The effect of the artifact placed to ABP only (bottom picture). The increased values of ABP due to the artifact placement do not cause the increase of PRx.
