# Supplementary material for: Effect of artifacts upon the pressure reactivity index
Source: Sci Rep. 2022 Sep 6;12:15131. doi: 10.1038/s41598-022-19101-y (PMC9448724; doi:10.1038/s41598-022-19101-y)
Supplement: Supplementary file 2 — Supplementary Table S1. [file 41598_2022_19101_MOESM2_ESM.docx]

Effect of Artifacts upon the Pressure Reactivity Index

- Supplementary material 2

Martin Rozanek, Josef Skola, Lenka Horakova, Valeriia Trukhan

**Supplementary table S1.** The ratio of Pressure Reactivity index (PRx) above the set threshold 0.3, observed following insertion of the modeled artifacts into undisturbed biosignals. Values are presented after insertion of artifacts only into arterial blood pressure (ABP) signal, or into both arterial blood pressure and intracranial pressure (ICP) signals. The colors indicate the change of PRx in minority (light grey) to majority (dark grey) of the biosignal segments. For the description of the artifact types, see Table 1, Fig. 1 and the results. The lengths and amplitudes of the individual artifacts were modeled based on the native artifacts.

| **Ratio of PRx above 0.3 for all artifact types (%)** | | | | | | | | | | | | | | | | | | | | | | | |
| --- | --- | --- | --- | --- | --- | --- | --- | --- | --- | --- | --- | --- | --- | --- | --- | --- | --- | --- | --- | --- | --- | --- | --- |
| Amplitude rise (%) | **Rectangular artifact (ABP)** | | | | | | | | | | | | **Rectangular artifact (ABP and ICP)** | | | | | | | | | | |
|  | Duration (s) | | | | | | | | | | | | Duration (s) | | | | | | | | | | |
|  | 4 | | 15 | | | | 30 | | | 60 | | | 4 | | 15 | | | | 30 | | | 60 | |
| 25 | 0 | | 0.71 | | | | 1.43 | | | 2.14 | | | 55.42 | | 95 | | | | 100 | | | 100 | |
| 50 | 0.63 | | 1.43 | | | | 2.14 | | | 2.14 | | | 90.83 | | 100 | | | | 100 | | | 100 | |
| 75 | 0.63 | | 1.43 | | | | 4.29 | | | 2.86 | | | 97.5 | | 100 | | | | 100 | | | 100 | |
| 100 | 1.34 | | 2.14 | | | | 4.29 | | | 2.86 | | | 100 | | 100 | | | | 100 | | | 100 | |
| **Fast impulse (ABP)** | | | | | | | | | | | | | **Fast impulse (ABP and ICP)** | | | | | | | | | | |
| Amplitude rise (%) | | | | | | | | | | | | | Amplitude rise (%) | | | | | | | | | | |
| 25 | | 50 | | 75 | | | | 100 | | | 125 | | 25 | 50 | | | 75 | | | 100 | | | 125 |
| 0 | | 0 | | 0 | | | | 0 | | | 0 | | 0 | 0 | | | 0 | | | 0 | | | 0 |
| Amplitude rise (%) | **Saw tooth (ABP)** | | | | | | | | | | | | **Saw tooth (ABP and ICP)** | | | | | | | | | | |
|  | Duration (s) | | | | | | | | | | | | Duration (s) | | | | | | | | | | |
|  | 30 | | | | 45 | | | | 90 | | | | 30 | | | 45 | | | | | 90 | | |
| 30 | 2.14 | | | | 2.14 | | | | 2.38 | | | | 100 | | | 100 | | | | | 100 | | |
| 60 | 4.29 | | | | 2.14 | | | | 2.9 | | | | 100 | | | 100 | | | | | 100 | | |
| Amplitude rise (%) | **Isoline drift (ABP and ICP)** | | | | | | | | | | | | | | | | | | | | | | |
|  | Duration (s) | | | | | | | | | | | | | | | | | | | | | | |
|  | 15 | | | | | 30 | | | | | | 60 | | | | | | 120 | | | | | |
| 15 | 0.71 | | | | | 1.43 | | | | | | 1.43 | | | | | | 7.84 | | | | | |
| 30 | 6.96 | | | | | 16.43 | | | | | | 21.58 | | | | | | 37.45 | | | | | |
